# Accounting for aetiology: can regional surveillance data alongside host biomarker-guided antibiotic therapy improve treatment of febrile illness in remote settings?

**DOI:** 10.12688/wellcomeopenres.14976.2

**Published:** 2019-08-05

**Authors:** Arjun Chandna, Lisa J. White, Tiengkham Pongvongsa, Mayfong Mayxay, Paul N. Newton, Nicholas P. J. Day, Yoel Lubell

**Affiliations:** 1 Mahidol-Oxford Tropical Medicine Research Unit, Bangkok, Thailand; 2 Department for Clinical Research, London School of Hygiene & Tropical Medicine, London, UK; 3 Centre for Tropical Medicine and Global Health, University of Oxford, Oxford, UK; 4 Savannakhet Provincial Health Department, Lao-Oxford-Mahosot Hospital-Wellcome Trust Research Unit, Savannakhet Province, Lao People's Democratic Republic; 5 Institute of Research and Education Development, University of Health Sciences, Vientiane, Lao People's Democratic Republic

**Keywords:** Febrile illness, aetiology, surveillance, biomarker, C-reactive protein, Southeast Asia, rural, cost-effectiveness

## Abstract

**Background**: Across Southeast Asia, declining malaria incidence poses a challenge for healthcare providers, in how best to manage the vast majority of patients with febrile illnesses who have a negative malaria test. In rural regions, where the majority of the population reside, empirical treatment guidelines derived from central urban hospitals are often of limited relevance. In these settings, health workers with limited training deliver care, often without any laboratory diagnostic support. In this paper, we model the impact of point-of-care C-reactive protein testing to inform the decision to prescribe antibiotics and regional surveillance data to inform antibiotic selection, and then simulate the subsequent impact on mortality from febrile illnesses, rooted in the real-world context of rural Savannakhet province, southern Laos.

**Methods**: Our model simulates 100 scenarios with varying quarterly incidence of six key pathogens known to be prevalent in rural Laos. In the simulations, community health workers either prescribe antibiotics in-line with current practice as documented in health facilities in rural Laos, or with the aid of the two interventions. We provide cost-effectiveness estimates for each strategy alone and then for an integrated approach using both interventions.

**Results**: We find that each strategy is predicted to be highly cost-effective, and that the combined approach is predicted to result in the biggest reduction in mortality (averting a predicted 510 deaths per year in rural Savannakhet, a 28% reduction compared to standard practice) and is highly cost-effective, with an incremental cost-effectiveness ratio of just $66 per disability-adjusted life year averted.

**Conclusions**: Substantial seasonal variation in the predicted optimal empirical antibiotic treatment for febrile illness highlights the benefits of up-to-date information on regional causes of fever. In this modelling analysis, an integrated system incorporating point-of-care host biomarker testing and regional surveillance data appears highly cost-effective, and may warrant piloting in a real-life setting.

## Introduction

There is a growing body of evidence that host biomarker tests, including commercially available low-cost point-of-care varieties, can help health workers identify patients with febrile illnesses who might benefit from antibiotic treatment ^1, 2^. These tests have the potential to improve rational antibiotic prescribing, increasing the proportion of patients with bacterial infections that receive antibiotics, and diminishing overall drug pressure through fewer antibiotic prescriptions for patients with viral infections ^3^.

However, these tests cannot inform the *selection* of an antibiotic. Thus, apart from the few diseases for which pathogen-specific point-of-care tests (POCTs) are available, for the overwhelming majority of patients with febrile illnesses, initial antimicrobial choice is empirical.

In low- and middle-income countries (LMICs) empirical treatment guidelines are often derived from sparse data collected at central urban hospitals, which may have little relevance to the rural settings where the majority of the population live. For example, in rural areas of Southeast Asia scrub typhus is a leading cause of hospitalisation, yet empirical management of fever in the community rarely includes an anti-Rickettsial antibiotic, even in areas where scrub typhus is known to be endemic ^4^. Furthermore, seasonal, longitudinal and spatial heterogeneity pose additional challenges to providing locally relevant and up-to-date empirical treatment recommendations ^5^. Finally, the emergence and spread of antimicrobial resistant (AMR) infections further complicates and constrains the selection of appropriate antibiotic treatment ^6^.

Providing health workers with relevant data on the causes of febrile illness in their area would increase the probability that effective antibiotic therapy is selected and could improve patient outcomes. Until recently, acquiring such data was challenging, due to limited microbiological laboratory capacity in rural regions of most LMICs. However, new multiplex molecular testing platforms, including fully automated options that require no sample processing ^7^, have been shown to be practical for use in resource-limited settings ^8, 9^. Although these platforms cannot resolve all challenges associated with performing diagnostic microbiology in resource-constrained settings, they require less training and infrastructure than standard platforms. In conjunction with appropriate support, placing these platforms in hospitals that currently lack the infrastructure to run fully-functioning microbiology laboratories could not only improve treatment of inpatients with suspected infections, but also improve care for the broader population, who seek care at peripheral clinics and community health posts within the catchment area of the regional hospital.

In this paper, we first explore the cost-effectiveness of such a system in terms of the costs and benefits for hospitalised patients with suspected infections. We then model the impact and cost-effectiveness this system could have in guiding empirical treatment in patients presenting to peripheral health facilities and community health posts in the geographical area served by the regional hospital. We compare current practice to the use of POC host biomarker-guided antibiotic therapy alone, the use of aetiological surveillance data alone and then an integrated system in which both approaches are combined. Our aim is to determine whether this approach warrants further investigation in a real-life setting, and not to provide a robust estimate of the potential magnitude of its impact. We use C-reactive protein (CRP) as an example host biomarker, as it is currently the most well characterised host biomarker in our region ^2, 10^, and low-cost POCTs are already available on the market ^11^.

## Modelling the cost-effectiveness of point-of-care multiplex polymerase chain reaction platforms for hospitalised patients

Multiplex polymerase chain reaction (PCR) platforms are increasingly available. They can test for dozens of target pathogens simultaneously, require limited training and infrastructure, and are deployable in resource-limited settings. Although sensitivity is inferior to traditional microbiological techniques, in hospitals that lack the infrastructure and laboratory capacity to reliably perform investigations such as blood cultures, these platforms can provide timely and potentially life-saving POC diagnoses to inform the management of severely ill, hospitalised patients.

A simple ‘back of the envelope’ calculation suggests that assuming a reduction of just one percentage point in case fatality rate (CFR) due to early diagnosis facilitating optimisation of empirical antimicrobial treatment, this could be a highly cost-effective strategy, even in the context of low-income countries ( Table 1). This excludes further potential cost-savings in terms of antibiotic use averted, including both their direct purchase costs and the subsequent, and often higher costs of AMR associated with their use ^12^.

**Table 1.** Cost-effectiveness of a point-of-care multiplex PCR diagnostic platform in the management of hospitalised patients with suspected infections.

	Units	Unit cost	Total cost	Notes
One-step multiplex PCR device	0.2	$35,000	$7,000	Assume 5-year useful life
Server and peripheral equipment	0.2	$5,000	$1,000	
Laptop	0.2	$1,200	$240	
Laboratory technician	12	$1,000	$12,000	
**Total annual capital and labour costs**			$20,240 ^a^	
**Samples per annum**	1825 ^b^			Assume 5 samples/day ^15^
Capital and labour cost per sample			$11 ^c^	a/b
Multiplex panel (one per sample)	1	$155	$155 ^d^	
Other consumables	1	$5	$5 ^e^	
**Total cost per sample**			$171	c+d+e
Three scenarios for CFR without PCR			5%, 7%, 10%	
Three scenarios for CFR with PCR			4%, 5%, 7%	
DALYs per death			50	WHO age-adjusted life expectancy, based on a median age of 21 in hospitalised inpatients from a fever study in rural Laos ^16^
**Cost/DALY in three comparative scenarios**			**$342, $171, $114**	

DALY, disability adjusted life year.

## Modelling the impact of dynamic empirical treatment recommendations and point-of-care C-reactive protein guided antibiotic therapy

There is, however, a potential additional benefit of such a system – providing regional and timely data on causes of fever that can then be used to improve empirical treatment decisions for patients elsewhere in the region, who are unable to attend the hospital themselves. It is well recognised that compliance with recommendation for referral in many LMICs is low, due to geographical, financial and social constraints ^13^, and that this is particularly problematic for common childhood illnesses, such as paediatric pneumonia ^14^.

Data could be distributed to community health workers (CHWs) through smartphones equipped with electronic decision-support tools, that provide pragmatic guidance as to optimal antibiotic selection, given POC CRP test results or simple-to-elicit clinical symptoms indicative of a bacterial infection ^17^, and accounting for background information on causes of fever from the regional hospital multiplex PCR platform.

## Methods

### Model development

We developed a model in R, simulating the impact and cost-effectiveness of such an integrated system in the context of 1,500 villages, each with a CHW in rural Savannakhet province, southern Laos ^16^. We first simulate the outcomes of febrile illness due to common infections in this population in the absence of treatment. We then estimate their outcomes if CHWs were supplied with antibiotics to dispense based on clinical judgement, and then evaluate the potential impact of POC CRP-guided antibiotic therapy and/or the use of regional surveillance data on causes of fever to guide antibiotic prescribing. Our model assumes the following:

- A population of 880,000 people served by CHWs in Savannakhet province, based on census figures from the Lao Statistics Bureau at the Ministry of Planning and Investment ^18^: 1,000,000 people in the province of whom approximately 120,000 live in the provincial capital;
- An overall incidence of febrile illness of 0.33 per person per annum, distributed by quarter to reflect seasonality, and based on regional estimates for the burden of febrile illness ^19^;
- That 36.6% of these fevers are cases of dengue (8.7%), scrub typhus (6.8%), influenza (6.4%), Japanese encephalitis (6.2%), leptospirosis (6.1%), or bacteraemia (2.4%), based on a cause of fever study in rural Laos, and including only confirmed mono-infections that had a prevalence of 5% or above ^16^;
- Interventions only affect outcomes in patients with these known pathogens. The other 63.4% of simulated patients with unknown causes of fever receive no benefit from the interventions, although the costs of the interventions are applied to their management;
- Patients with malaria (1.1% in the original study) are excluded from the simulation as these would be managed based on a positive rapid diagnostic test (RDT) for malaria;
- The model runs 100 simulated scenarios with a stochastically determined incidence of the above diseases, drawn from gamma distributions with means shown in Table 2, reflecting the uncertainty and heterogeneity in their incidence;
- Viral infections convey a CFR of 0.1% irrespective of receiving an antibiotic ^20, 21^;
- CFRs for scrub typhus in the absence of a tetracycline are 6% ^22^, for leptospirosis it is 2.2% in the absence of either a tetracycline or a beta-lactam ^23^, and for bacteraemia in the absence of a beta-lactam the CFR would be 15% ^24, 25^;
- Deaths are associated with a loss of life of 50 years as described in Table 1, conservatively based on World Health Organization (WHO) age-adjusted life tables ^27^;
- To classify an intervention as cost-effective we compare its incremental cost-effectiveness ratio (ICER) per disability-adjusted life year (DALY) averted to a conservative willingness to pay (WTP) threshold of $1230, half the Laos Gross Domestic Product (GDP) per capita ^28^;
- Healthcare workers in the field must choose between providing no treatment, a beta-lactam, or a tetracycline antibiotic. In the absence of CRP-testing or the surveillance data, their prescribing practices are conservatively assumed to resemble those of more highly-trained healthcare workers in the fever study from Laos ^16^, detailed in Table 2, whereby for example 26% of patients with scrub typhus received a tetracycline;
- POC CRP tests with a threshold of 40mg/L to guide the decision to prescribe (CRP ≥ 40mg/l) or withhold (CRP < 40mg/l) antibiotics, have a sensitivity and a specificity as detailed by pathogen in Table 2, based on data from prospective studies conducted in Cambodia, Laos and Thailand ^2^;
- An incremental cost of $2 per POC CRP test;
- The costs of establishing a multiplex PCR platform for regional surveillance is $42,200 with an expected useful life for the equipment of 5 years, and a cost of reagents and consumables per specimen of $160 (as detailed in Table 1);
- We assume that a subset of 1/50 samples from patients with febrile illnesses in the community would be collected for multiplex PCR testing to inform the regional estimates for causes of fever (i.e. we conservatively assume that all costs incurred are above and beyond those that would be incurred if the platform was installed for use in hospitalised patients and regional estimates obtained from the diagnoses in these patients).

**Table 2.** Model parameters.

Disease	Proportion	CFR when untreated	Effective antibiotic	Standard practice (No treatment, beta- lactam, tetracycline)	Probability CRP high
**Dengue**	8.7%	0.1%	-	41%, 41%, 18%	12%
**Scrub typhus**	7.9%	6%	Tetracycline	34%, 39%, 26%	70%
**Influenza**	6.3%	0.1%	-	64%, 32%, 4%	20%
**JEV**	6.2%	0.1%	-	27%, 63%, 10%	42%
**Leptospirosis**	6.1%	2.2%	Tetracycline or beta- lactam	34%, 39%, 26%	81%
**Bacteraemia**	2.4%	15%	Beta-lactam	43%, 33%, 23%	84%
**Unconfirmed**	62.4%	0.5%	-	49%, 38%, 13%	36%
**Notes/sources**	16	20– 25, 26	16		2

Using these assumptions and parameter estimates we compare the population mortality rates and costs for the following strategies, as compared with a hypothetical situation of no treatment:

1) Standard prescribing practices based on those documented at rural health facilities in Laos ^16^
2) POC CRP-guided antibiotic therapy, with decision to prescribe antibiotics determined by a POC CRP-test with the above assumed test characteristics, and subsequent antibiotic selection in proportion to that in standard practice
3) The decision on whether to prescribe an antibiotic equates to that in standard practice, but the selection of antibiotic is based on the regional surveillance data, using an algorithm that selects the antibiotic with the best expected value in terms of health gains given the known incidence of infections in the region at that point in time (i.e. min∑(incidence[i]*CFR[i]*(1-efficacy[a]), where [i] relates to each of the pathogens in that quarter and iteration of the simulation and [a] is each of the two antibiotics). The model allows for the fact that the random selection of samples from 1/50 patients might not be a true reflection of the actual distribution of pathogens in the broader population, in which case the recommended empirical treatment will not be optimal
4) A combination of both approaches, in which decision to prescribe antibiotic is determined by the POC CRP test and subsequent selection of antibiotic determined by the above regional surveillance data algorithm.

This is repeated in 100 simulations to capture the heterogeneity in causes of fever. The mean expected mortality under each of the four strategies is then compared with a hypothetical situation of no treatment.

### Cost-effectiveness estimation

The total mortality for the population of 880,000 is converted to DALYs. The four strategies are then compared against a hypothetical situation of no treatment, and ranked in order of effectiveness. The incremental costs and gains are then used to estimate the ICER per DALY averted, from least effective to most effective, to identify strategies that are strongly dominated (i.e. both more costly and less effective than other options). This process is repeated to identify strategies that are weakly dominated (i.e. there are more effective strategies with lower ICERs). The ICERs for the remaining strategies are then re-estimated and plotted as a cost-effectiveness frontier on the cost-effectiveness plane. We also plot cost-effectiveness acceptability curves, showing the proportion of iterations in which each strategy was identified as most cost-effective at varying levels of WTP per DALY averted.

## Results

### Predicted number of deaths

**Figure 1.**
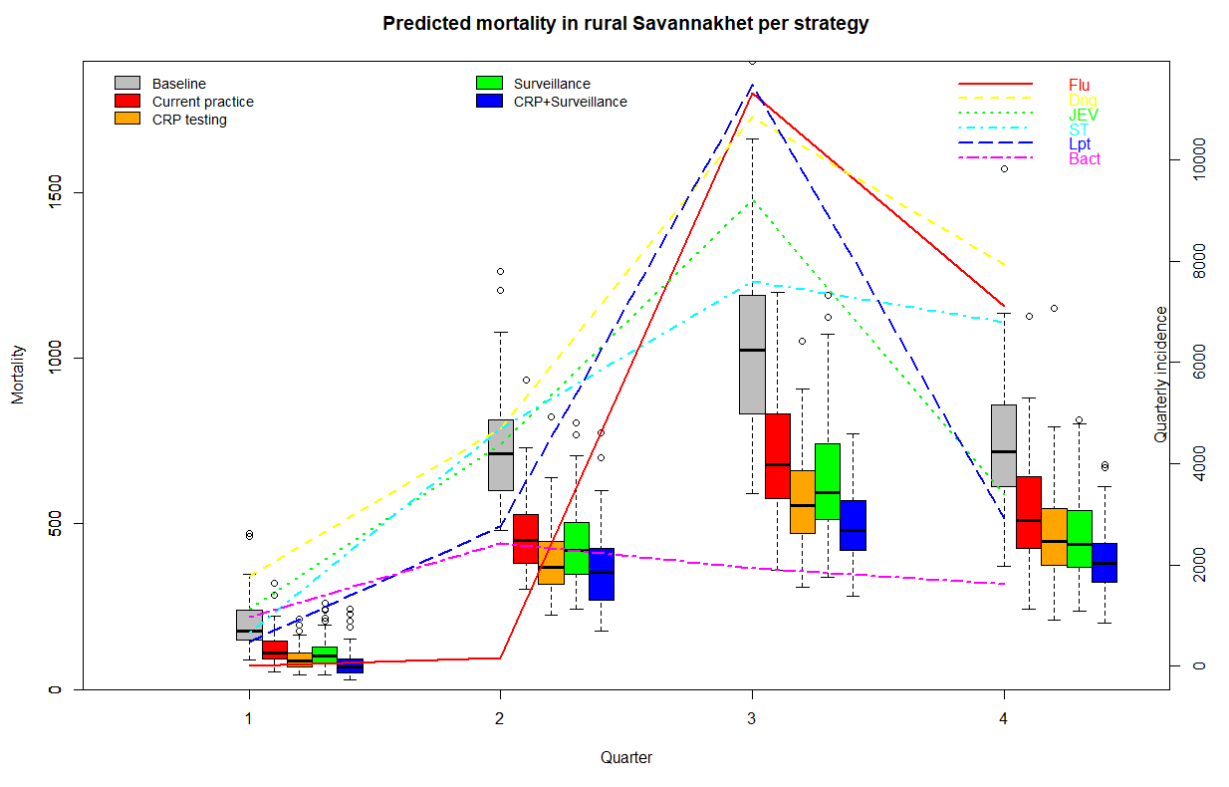
Predicted mortality under each treatment strategy and predicted incidence of the six pathogens, by quarter.

In the absence of any treatment, the model predicts 2701 deaths per year in the rural population of Savannakhet (equivalent to a mortality rate of 307 deaths per 100,000 person-years) due to the six pathogens. Current antibiotic prescribing practice would avert 863 of these deaths. The use of CRP-guided antibiotic therapy, without the surveillance data, would avert an additional 325 deaths due to identification of a greater proportion of patients with bacterial infections whom may benefit from antibiotic treatment. Use of the regional surveillance data alone would have a lower impact than CRP-guided antibiotic therapy alone, reducing mortality by an additional 192 deaths compared with current prescribing practice. The combined regional surveillance data and CRP-guided antibiotic strategy was predicted to avert most deaths, achieving reductions of 510 deaths compared with current practice, or 1373 deaths compared with the hypothetical situation of no treatment. The distribution of these deaths over the entire year are shown in Table 3 and by quarter are shown in Figure 1.

**Table 3.** Predicted number of deaths in rural Savannakhet under each strategy.

Strategy	Predicted deaths in rural Savannakhet, n (95% CI)
No treatment	**2,700** (2,590-2,820)
Standard practice	**1,840** (1,760-1,930)
Surveillance-guided treatment	**1,650** (1,580-1,720)
CRP-guided treatment	**1,510** (1,440-1,590)
CRP- and surveillance-guided treatment	**1,330** (1,270-1,390)

### Predicted treatment choices

The overall proportion of patients prescribed an antibiotic was 15 percentage points lower in the two strategies that included CRP-guided antibiotic therapy than those without, at 44% as compared with 59%. The proportion of 100 simulated scenarios in which either a beta-lactam or a tetracycline would be recommended by the surveillance system, broken down by each quarter, is shown in Table 4. There is much variability in the optimal treatment choice between and within the four quarters over the 100 simulated scenarios, illustrating the importance of up-to-date surveillance data.

**Table 4.** Percentage of the time that either a beta-lactam or a tetracycline would be recommended by the surveillance system, in each quarter.

Drug	Quarter 1	Quarter 2	Quarter 3	Quarter 4
**Beta-lactam**	98%	75%	24%	9%
**Tetracycline**	2%	25%	76%	91%

### Predicted costs and cost-effectiveness

The incremental costs of deploying the CRP tests as compared with standard practice were estimated at $505,000, less than half the estimated incremental cost of the regional surveillance system, which was $1,162,000. Given these costs and benefits, the ICER per DALY averted for the CRP-guided antibiotic therapy strategy alone compared with current practice was $32, while the ICER per DALY averted for the regional surveillance system alone compared with current practice was $121, therefore individually they can both be considered very cost-effective.

**Figure 2.**
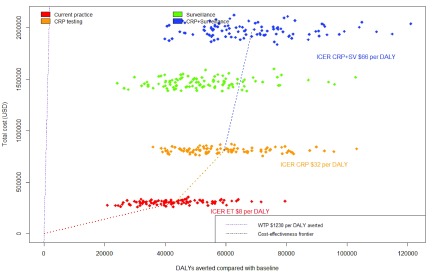
The simulated costs and benefits of the four alternative strategies.

In a multi-way comparison the use of the regional surveillance data alone was excluded due to being strongly dominated by the CRP-guided antibiotic therapy strategy; the ICER of the combined strategy of CRP-guided antibiotic therapy and regional surveillance data as compared with CRP-guided antibiotic therapy alone was $66, therefore highly cost-effective. The simulated costs and benefits of each strategy across the 100 simulations and the cost-effectiveness frontier are shown in Figure 2.

**Figure 3.**
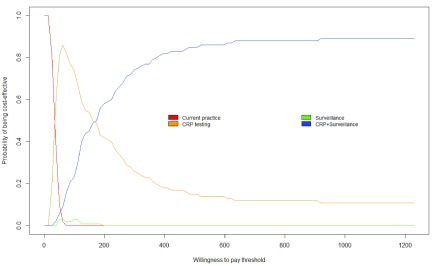
Cost-effectiveness acceptability curves for the four strategies.

The cost-effectiveness acceptability curves for the four strategies is shown in Figure 3. This indicates that at a WTP of over $30 per DALY averted there is a greater than 50% probability of the CRP-guided antibiotic therapy strategy being most cost-effective, while the combined strategy is most likely to be cost-effective at a WTP value of over $200 per DALY averted, and this approaches 100% at a WTP threshold of $600, well below the conservative threshold of $1230 per DALY averted, half of the Laos GDP per capita.

## Discussion

After decades of presumptive antimalarial treatment for patients with febrile illness in endemic settings, the decline in malaria transmission and the widespread availability of malaria RDTs poses a challenge to healthcare providers in how best to manage the vast majority of patients with febrile illnesses who have a negative malaria test.

This study models the impact and cost-effectiveness of introducing host biomarker testing to guide the decision to prescribe antibiotics, and regional surveillance data to guide the selection of antibiotic when they are thought to be required. In isolation, both CRP-guided antibiotic therapy and the use of regional surveillance data were cost-effective, although CRP-guided antibiotic therapy was both less costly and more effective than the surveillance system. The combination of the two interventions was the most cost-effective option, delivering the largest reductions in mortality (28% as compared with current practice), with an ICER of just $66 per DALY averted.

To capture any variation in results in response to spatial and longitudinal heterogeneity in causes of fever, we ran the model over 100 simulated scenarios with different pathogen incidences. In all these scenarios the relative gains and cost-effectiveness estimates were largely consistent, with the combined strategy being the most effective, and both CRP-guided antibiotic therapy and surveillance data alone being more effective than current practice in 100% of scenarios, and the CRP-guided treatment being more effective than surveillance strategy alone in 77% of scenarios.

Our estimate is deliberately conservative. As previously stated our aim was to determine whether this approach warrants further investigation in a real-life setting, and not to provide a robust estimate of the potential magnitude of its impact. In fever studies from the region (and elsewhere), a pathogen is detected in only a minority of patients ^16^. Given the known limitations in sensitivity of all currently available diagnostics, it is likely that some patients in whom the cause of fever remains unknown have a bacterial infection that may benefit from treatment with antibiotics. Indeed CRP levels in patients without a known cause for their infection are on average higher than those with an identified viral infection ^2^. In this modelling simulation, the majority of patients were not assigned a specific pathogen, and we assumed no health benefits conveyed by antibiotics (prescribed empirically or with the guidance of CRP-testing) in these patients. It is likely therefore that the results are an underestimate of the potential health gains associated with this approach.

In addition, by calculating DALYs, we considered only benefit conveyed by preventing years of life lost in the small number of deaths, but not from any reduction in the duration of time that patients were ill and potentially unable to work or attend school. Furthermore, we did not include the cost benefits of a 15% reduction in antibiotic prescription rates, neither in terms of direct purchasing costs nor avoidance of AMR. Including these benefits would increase the cost-effectiveness of both strategies that utilise CRP-guided antibiotic therapy.

Our study benefits from being rooted in the real-world context of Savannakhet province, with the majority of model assumptions informed by prospectively collected regional data. However, we recognise a number of limitations. The performance characteristics of POC CRP tests with a threshold of 40 mg/l are informed by prospective studies conducted by our research unit, predominantly recruiting outpatients with non-severe illnesses. We are aware of the limitations of using CRP-testing to guide antimicrobial therapy in all patients with febrile illness, particularly with regards lack of sensitivity in patients with severe illnesses ^29^. We recognise the need for a robust assessment of illness severity to precede any potential use of POC CRP-guided antibiotic therapy, if the approach we have explored in this paper is to be piloted in a real-life setting.

We assume that appropriate treatment (for example, a beta-lactam for bacteraemia), is 100% effective. Similarly, we assume that inappropriate treatment (for example, a tetracycline for bacteraemia) has no effect on patient outcome. This ‘all or nothing’ approach is simplistic and does not account for inter-patient heterogeneity, for example idiosyncratic bioavailability of antibiotics or patient comorbidities, adjunctive therapies in addition to antibiotics (for example, referral to higher-level care) and other host factors that may contribute to pathogenesis and response to treatment. Nor does it account for the need to provide intravenous antibiotic treatment, for example in those unable to take oral medications or in patients with melioidosis or multi-drug resistant infections where oral treatment options may not be available.

A further simplification is the inclusion of only two classes of antibiotics—beta-lactams and tetracyclines. This was a deliberate decision, taken to simulate the probable scenario that a limited-skill CHW can only be expected to reliably dispense one or two different antibiotics. In the original Laos fever study ^14^ beta-lactams and tetracyclines made up 81% of all prescriptions. Firstly, we assumed that each patient received only one class of antibiotic. Secondly, we conservatively allocated the remaining 19% of prescriptions to the beta-lactam group; scrub typhus (tetracycline-sensitive) was more prevalent than bacteraemia (beta-lactam-sensitive) in this simulated cohort, hence this approach will have reduced our cost-effectiveness estimates, compared with the alternative of allocating some or all the additional 19% of prescriptions to the tetracycline group.

Empirical treatment recommendations are determined by the simulated relative proportions of different bacterial pathogens, amongst patients in whom a cause of fever is determined. Only pathogens that were diagnosed (and by definition, tested for) in the original Laos fever study are included in our model. Hence the spectrum of simulated pathogens is necessarily determined by the comprehensiveness of the original diagnostic panel. It is therefore conceivable that a more comprehensive diagnostic panel could alter the proportional incidence of particular pathogens (potentially including additional pathogens). For example, a high prevalence of Streptococcal ** disease, not specifically tested for in the original fever study, amongst patients with acute respiratory infections, the most common presenting syndrome in patients with febrile illness, could make selection of a beta-lactam antibiotic always the optimal choice. This would make the contribution of a regional surveillance system, in terms of guiding empirical antibiotic therapy, redundant. Whilst we do not feel that this scenario is likely, a prospective study with an exhaustive aetiological diagnostic panel would be required to conclusively reject this possibility.

In addition, the accuracy of our simulated pathogen spectrum is limited by the fact that influenza testing only occurred during one of the three ‘flu seasons’, and at only one of the two hospitals in the original study. We decided not to extrapolate these results to the whole study period. Doing so would have increased the relative proportion of influenza cases and improved our cost-effectiveness estimates by virtue of the fact that more patients would have been assigned a simulated diagnosis of influenza, the lowest CFR in the model (0.1%) and fewer patients assigned diagnoses of scrub typhus, leptospirosis, bacteraemia or unknown, all of which have higher CFRs. It should be noted that whilst our CFRs are informed by the available literature, they will inevitably be affected by ascertainment bias of patients with more severe disease, and will therefore, in general, be overestimates.

In our model empirical treatment recommendations are updated on a quarterly basis in response to the results of diagnostic investigations performed on random samples taken from patients with febrile illness attending CHWs. We allow for the fact that the number of samples taken in the community might not be sufficiently large to consistently represent the actual distribution of detected pathogens in the broader population. This could of course be improved with a higher sampling frequency (at a higher cost). The model, however, did not account for imperfect sensitivities of the multiplex molecular testing platform, and the degree to which the sampling distribution represents the true population distribution of pathogens will be influenced by the sensitivity of the diagnostic investigations. It is likely that samples from remote areas would be restricted to small volume dried blood specimens, and hence diagnostic yield may be low. Further work is required to better understand the utility of the approach proposed here and whether sampling from patients with febrile illnesses attending rural clinics may provide more accurate representation of community fever aetiology, by virtue of the fact that larger sample volumes may improve diagnostic yield.

Standard prescribing practices are assumed to reflect that in the original Laos fever study, which recruited both inpatients and outpatients. It is feasible that the cohort of patients that attend hospital may not be reflective of those that consult a CHW: patients with more severe disease may choose to seek care at a hospital whilst patients with fulminant disease may be underrepresented as they may die before reaching a health facility. This highlights an inherent challenge of using health facility-based presentations to infer community-level epidemiology. As outlined above, whilst population-based surveillance (for example, collecting samples directly from patients presenting to CHWs) appears attractive, further work is required to determine operational feasibility and the impact that low volume dried blood specimens may have on diagnostic yield.

Assuming equivalence in prescribing practices for patients presenting to CHWs and those presenting to hospital in the original study, may have overestimated antibiotic prescription rates amongst CHWs in the simulated standard practice scenarios. However, we believe it is reasonable to assume that, without guidance, CHWs would indeed prescribe at higher rates than their higher-trained counterparts. In addition, CHW prescriptions based on clinical judgement are likely to be less well targeted against the underlying pathogen. We have not accounted for this in our model and doing so would improve the cost-effectiveness of the surveillance system, compared to standard practice.

The estimated fever incidence of 0.33 episodes per person per annum is taken from a multi-country (Indonesia, Malaysia, Philippines and Thailand) observational cohort study ^19^ in children aged 2 to 14 years, which used a robust definition of fever (documented axillary temperature of ≥38°C). It is possible that this estimate may not reflect the burden of febrile illness in Savannakhet province. However, the estimate is broadly consistent with fever incidence from CHW programmes in Southeast Asia, with whom our research unit collaborate.

Finally, an inherent assumption in the proposition of an integrated regional surveillance system like the one we discuss in this paper, is that adequate pathogen-specific POCTs are not yet available for common circulating pathogens. In our context, a RDT for scrub typhus has great potential to improve patient management. Whilst a regional surveillance system can indicate in which geographical areas a tetracycline should be first-line treatment for febrile illness, a scrub typhus RDT could indicate which individual patients would be most likely to benefit from this treatment. Current scrub typhus RDTs are affected by high background seroprevalence in endemic areas ^30^. If an affordable antigen-based scrub typhus RDT with acceptable reliability and validity is developed, a surveillance system like the one we propose, could help guide in which areas and in which seasons it might most usefully be deployed.

## Conclusions

Treatment of febrile illness, and specifically the decision on whether and which antibiotics are warranted is challenging even for experienced clinicians. In rural areas of LMICs where frontline healthcare workers have limited training and no laboratory support, antibiotic targeting is understandably poor. Tools to improve this are, however, available and deploying them could reduce an avoidable burden of illness and overuse of antibiotics.

A previous modelling analysis undertaken by our group found CRP-guided antibiotic therapy alone to be cost-effective in the management of non-malarial febrile illness in rural Laos ^3^. Here we took a very different modelling approach to consider CRP-guided antibiotic therapy alongside the use of regional surveillance data on causes of fever to provide dynamic empirical treatment recommendations for patients with febrile illness. In this modelling analysis this approach appears highly cost-effective, and may warrant piloting in a real-life setting.

## Data availability

All data underlying the results are available as part of the article and no additional source data are required.

## Software availability

The model code is available for download: DOI: https://doi.org/10.5281/zenodo.2206939 ^31^.

License: Creative Commons Attribution 4.0 International (CC BY 4.0).

## References

[1] Althaus T Greer RC Swe MMM: Effect of point-of-care C-reactive protein testing on antibiotic prescription in febrile patients attending primary care in Thailand and Myanmar: an open-label, randomised, controlled trial. *Lancet Glob Health.* 2019;7(1):e119–e131. 10.1016/S2214-109X(18)30444-3 30554748 PMC6293968

[2] Lubell Y Blacksell SD Dunachie S: Performance of C-reactive protein and procalcitonin to distinguish viral from bacterial and malarial causes of fever in Southeast Asia. *BMC Infect Dis.* 2015;15:511. 10.1186/s12879-015-1272-6 26558692 PMC4642613

[3] Lubell Y Althaus T Blacksell SD: Modelling the Impact and Cost-Effectiveness of Biomarker Tests as Compared with Pathogen-Specific Diagnostics in the Management of Undifferentiated Fever in Remote Tropical Settings. *PLoS One.* 2016;11(3):e0152420. 10.1371/journal.pone.0152420 27027303 PMC4814092

[4] Wangrangsimakul T Althaus T Mukaka M: Causes of acute undifferentiated fever and the utility of biomarkers in Chiangrai, northern Thailand. *PLoS Negl Trop Dis.* 2018;12(5):e0006477. 10.1371/journal.pntd.0006477 29852003 PMC5978881

[5] White LJ Newton PN Maude RJ: Defining disease heterogeneity to guide the empirical treatment of febrile illness in resource poor settings. *PLoS One.* 2012;7(9):e44545. 10.1371/journal.pone.0044545 23028559 PMC3448597

[6] Zellweger RM Carrique-Mas J Limmathurotsakul D: A current perspective on antimicrobial resistance in Southeast Asia. *J Antimicrob Chemother.* 2017;72(11):2963–72. 10.1093/jac/dkx260 28961709 PMC5890732

[7] Hanson KE Couturier MR: Multiplexed Molecular Diagnostics for Respiratory, Gastrointestinal, and Central Nervous System Infections. *Clin Infect Dis.* 2016;63(10):1361–7. 10.1093/cid/ciw494 27444411 PMC5091344

[8] Eibach D Krumkamp R Hahn A: Application of a multiplex PCR assay for the detection of gastrointestinal pathogens in a rural African setting. *BMC Infect Dis.* 2016;16(1):150. 10.1186/s12879-016-1481-7 27080387 PMC4832549

[9] Leski TA Ansumana R Taitt CR: Use of the FilmArray System for Detection of *Zaire ebolavirus* in a Small Hospital in Bo, Sierra Leone. *J Clin Microbiol.* 2015;53(7):2368–70. 10.1128/JCM.00527-15 25972415 PMC4473222

[10] Aabenhus R Jensen JU Jørgensen KJ: Biomarkers as point-of-care tests to guide prescription of antibiotics in patients with acute respiratory infections in primary care. *Cochrane Database Syst Rev.* 2014(11):CD010130. 10.1002/14651858.CD010130.pub2 25374293

[11] Foundation for Innovative New Diagnostics.CRP Landscape.2017.

[12] Shrestha P Cooper BS Coast J: Enumerating the economic cost of antimicrobial resistance per antibiotic consumed to inform the evaluation of interventions affecting their use. *Antimicrob Resist Infect Control.* 2018;7(1):98. 10.1186/s13756-018-0384-3 30116525 PMC6085682

[13] al Fadil SM Alrahman SH Cousens S: Integrated Management of Childhood Illnesses strategy: compliance with referral and follow-up recommendations in Gezira State, Sudan. *Bull World Health Organ.* 2003;81(10):708–16. 14758430 PMC2572328

[14] Chowdhury EK El Arifeen S Rahman M: Care at first-level facilities for children with severe pneumonia in Bangladesh: a cohort study. *Lancet.* 2008;372(9641):822–30. 10.1016/S0140-6736(08)61166-6 18715634

[15] Hantrakun V Somayaji R Teparrukkul P: Clinical epidemiology and outcomes of community acquired infection and sepsis among hospitalized patients in a resource limited setting in Northeast Thailand: A prospective observational study (Ubon-sepsis). *PLoS One.* 2018;13(9):e0204509. 10.1371/journal.pone.0204509 30256845 PMC6157894

[16] Mayxay M Castonguay-Vanier J Chansamouth V: Causes of non-malarial fever in Laos: a prospective study. *Lancet Glob Health.* 2013;1(1):e46–54. 10.1016/S2214-109X(13)70008-1 24748368 PMC3986032

[17] Keitel K Kagoro F Samaka J: A novel electronic algorithm using host biomarker point-of-care tests for the management of febrile illnesses in Tanzanian children (e-POCT): A randomized, controlled non-inferiority trial. *PLoS Med.* 2017;14(10):e1002411. 10.1371/journal.pmed.1002411 29059253 PMC5653205

[18] Ministry of Planning and Investment LP Laos Statistics Bureau: Statistical Yearbook 2017.2018.

[19] Capeding MR Chua MN Hadinegoro SR: Dengue and other common causes of acute febrile illness in Asia: an active surveillance study in children. *PLoS Negl Trop Dis.* 2013;7(7):e2331. 10.1371/journal.pntd.0002331 23936565 PMC3723539

[20] Hadler JL Konty K McVeigh KH: Case fatality rates based on population estimates of influenza-like illness due to novel H1N1 influenza: New York City, May-June 2009. *PLoS One.* 2010;5(7):e11677. 10.1371/journal.pone.0011677 20657738 PMC2908148

[21] World Health Organization: Dengue: Guidelines for diagnosis, treatment, prevention and control.2009 Reference Source 23762963

[22] Taylor AJ Paris DH Newton PN: A Systematic Review of Mortality from Untreated Scrub Typhus (Orientia tsutsugamushi). *PLoS Negl Trop Dis.* 2015;9(8):e0003971. 10.1371/journal.pntd.0003971 26274584 PMC4537241

[23] Taylor AJ Paris DH Newton PN: A Systematic Review of the Mortality from Untreated Leptospirosis. *PLoS Negl Trop Dis.* 2015;9(6):e0003866. 10.1371/journal.pntd.0003866 26110270 PMC4482028

[24] Butler T Knight J Nath SK: Typhoid fever complicated by intestinal perforation: a persisting fatal disease requiring surgical management. *Rev Infect Dis.* 1985;7(2):244–56. 10.1093/clinids/7.2.244 3890097

[25] White NJ Dance DA Chaowagul W: Halving of mortality of severe melioidosis by ceftazidime. *Lancet.* 1989;2(8665):697–701. 10.1016/S0140-6736(89)90768-X 2570956

[26] Iuliano AD Roguski KM Chang HH: Estimates of global seasonal influenza-associated respiratory mortality: a modelling study. *Lancet.* 2018;391(10127):1285–300. 10.1016/S0140-6736(17)33293-2 29248255 PMC5935243

[27] Life Tables by Country: Laos PDR.2018; (Accessed: 29th October 2018). Reference Source

[28] Ochalek J Lomas J Claxton K: Cost per DALY averted thresholds for low- and middle-income countries: evidence from cross country data.2017 10.7490/f1000research.1113912.1

[29] Carrol ED Mankhambo LA Jeffers G: The diagnostic and prognostic accuracy of five markers of serious bacterial infection in Malawian children with signs of severe infection. *PLoS One.* 2009;4(8):e6621. 10.1371/journal.pone.0006621 19675669 PMC2721152

[30] Saraswati K Day NPJ Mukaka M: Scrub typhus point-of-care testing: A systematic review and meta-analysis. *PLoS Negl Trop Dis.* 2018;12(3):e0006330. 10.1371/journal.pntd.0006330 29579046 PMC5892940

[31] YLubell: YLubell/SEA-CTN: surveillance-poct (Version v1). 2018 10.5281/zenodo.2206940

