# Supplementary material for: Accounting for aetiology: can regional surveillance data alongside host biomarker-guided antibiotic therapy improve treatment of febrile illness in remote settings?
Source: Wellcome Open Res. 2019 Aug 5;4:1. Originally published 2019 Jan 2. [Version 2] doi: 10.12688/wellcomeopenres.14976.2 (PMC6589932; doi:10.12688/wellcomeopenres.14976.2)
Supplement: Peer review history

# Peer review: Reviewer response for version 1

**DOI:** 10.21956/wellcomeopenres.16335.r35679

**Published:** 2019-06-21

**Authors:** Ross M. Boyce

**Affiliations:** 1 Division of Infectious Diseases, University of North Carolina at Chapel Hill, Chapel Hill, NC, USA

**Summary:**

 The submitted manuscript describes the simulated impact and cost-effectiveness of various strategies, including the use of point-of-care biomarker testing and regional surveillance data - as derived from the implementation of forwardly-deployed multiplex PCR - to guide antibiotic therapy in a resource-limited setting. Data informing the model are derived from previous work in SE Asia by this well-established research group. This issue, namely the accurate and timely diagnosis and treatment of non-malarial febrile illness, is incredibly important to advancing many of the Sustainable Development Goals, and the approach the authors have taken is an important preliminary step in developing effective and feasible interventions.

 The manuscript is well organized, clearly written, and highly readable. In particular, I would commend the authors for the detailed and transparent description of the assumptions and methods underpinning the simulations. The included tables and figures are appropriate and well-designed. The results of the study are compelling and certainly suggest that additional prospective studies are warranted. The finding that CRP-guided antibiotic prescribing led to a decrease in deaths due to the identification of a greater proportion of patients with bacterial infections was especially notable, as much of the existing literature emphasizes the potential for biomarkers to reduce unnecessary antibiotic use. This is an important, albeit simulated, finding. 

 Overall, I have few criticisms and/or comments, which I have outlined below:

**Major Comments:**

1. The cost inputs for the implementation of a multiplex PCR infrastructure (Table 1) do not account for the analysis of surveillance data on a quarterly basis. I assume that this would require basic epidemiological analysis skillsets that, based on my experience in East Africa, are often not available at district-level hospitals. Even when there is a health information manager at the district level, these individuals are often overburdened with routine reporting requirements. I am somewhat skeptical that this type of work could be conducted on a quarterly basis without an increase in human resources. Similarly, there are no inputs to reflect the cost of dissemination, which I assume would be done through meetings with CHWs or software updates to mHealth platforms. I do not think this negates the principle study findings, but I suspect there is more complexity and cost to the proposed interventions than accounted for in the analysis. 
2. The assumption of case fatality rates for all viral diseases seems particularly low to me, especially for pediatric dengue and influenza, which are assumed to be highly incident in the model cohort. Such a low rate may be reasonable for viral pathogens that cause many upper respiratory infections (i.e. rhinovirus, coronavirus), but less so for dengue and influenza. 
3. It would be interesting to see different analyses for children and adults as one would expect differences in (i) the incidence of febrile illness, (i) epidemiology of disease, and (iii) case fatality rates. 

**Minor Comments:**

 Modeling the cost-effectiveness… 

- 1 ^st ^Paragraph: While multiplex PCR platforms require less training and infrastructure than standard platforms, they are not without their own issues such as routine maintenance and problem-solving. Depending on the platform, heat and/or dust can be adversely impact performance. Of course, problems with power-interruptions and the weak logistical supply chains remain.

Modeling the impact… 

- 2 ^nd ^Paragraph: Perhaps worth referencing Keitel *et al*. *(*2017) ^1^ that adopted a similar approach using an electronic clinical decision algorithm (Note – I am not affiliated with the authors of this study in any way).

 Methods 

- While the burden of malaria is quite low in this study, there remains the issue of asymptomatic parasitemia, which may or may not be detected by RDT and may or may not be contributing to the febrile illness. I don’t think this needs to be addressed here, but worthwhile considering given that some individuals with a positive malaria RDT may not receive required antibiotics. See recent manuscript by Dalrymple *et al*. **(2019) ^2^.

 Results 

- Is there any data on routine vital statistics from the study area that could be used to validate the estimate of 2,701 deaths per year and the prevention of 863 deaths with current practice?

 Discussion: 

- While tetracyclines are generally well-tolerated, they are often not used in pregnant women and young children. I am not familiar with recommended alternative regimens for scrub typhus (if any) and whether there would be any expected increase in adverse events associated with increased prescription of this class of antibiotics.

I confirm that I have read this submission and believe that I have an appropriate level of expertise to confirm that it is of an acceptable scientific standard.

## References

[1] : A novel electronic algorithm using host biomarker point-of-care tests for the management of febrile illnesses in Tanzanian children (e-POCT): A randomized, controlled non-inferiority trial. *PLoS Med*.2017;14(10) : 10.1371/journal.pmed.1002411 e1002411 10.1371/journal.pmed.1002411 29059253PMC5653205

[2] : The contribution of non-malarial febrile illness co-infections to Plasmodium falciparum case counts in health facilities in sub-Saharan Africa. *Malar J*.2019;18(1) : 10.1186/s12936-019-2830-y 195 10.1186/s12936-019-2830-y 31186004PMC6560910

# Response

**Published:** 2019-07-30

**Authors:** Arjun Chandna

**Affiliations:** Mahidol-Oxford Tropical Medicine Research Unit, Thailand

We thank the reviewer for their comprehensive comments on our manuscript and we are pleased that the reviewer recognises the need for strategies to improve care for patients with non-malarial febrile illnesses. Below we respond to the reviewer’s specific comments.

We agree that a basic epidemiological skillset would be required to analyse the quarterly data and determine whether and how empirical treatment guidelines should be updated. We did not account for these costs nor the costs for disseminating results and implementing updates to treatment guidelines, but a sensitivity analysis exploring the impact of higher costs for the surveillance system indicates that these have a negligible impact (for brevity we opted not to include this in the manuscript, although interested readers can explore these and other parameter estimates using the published code). As the reviewer describes, with advances in information and communication technologies it is feasible that these activities (the data analysis and dissemination) could be centralised, with results being delivered to mHealth platforms. A need would still remain for trained personnel at each health facility with whom central staff could liaise and to act as a focal point for local health workers. This human interaction is crucial to foster trust and achieve behaviour change. We acknowledge that including these additional costs would improve the accuracy of our cost-effectiveness estimates, but as the reviewer mentions, their omission is unlikely to alter the principle findings.

It is challenging to determine accurate case fatality rates (CFR) for febrile illnesses, especially for viral infections, as a substantial proportion are asymptomatic or of insufficient severity to trigger care seeking, particularly to central levels of the health system, where surveillance activities are often conducted (and estimates for CFRs calculated). Our CFRs for viral infections are based on the available literature: a CFR of between 0.05 to 0.09% for H1N1 influenza in New York City, established using active surveillance (i.e. telephone surveys); and the World Health Organization’s estimate of 0.2 to ~1.0% for the CFR for dengue in Southeast Asia, using passive surveillance (i.e. only patients severe enough to present to a level of healthcare where diagnostic tests are present contribute to the denominator). Hence overall, we believe our estimate to be conservative, and the actual CFRs for illnesses due to these pathogens presenting at the community health worker level are likely to be substantially lower. Notwithstanding, motivated by the reviewer's comments, we ran a sensitivity analysis using an exceedingly high CFR for viral infections of 1% and while this resulted in much higher overall mortality in the simulated cohort, this had no impact on the cost-effectiveness of both host biomarker- and surveillance-guided strategies (alone or in combination) as these are determined by the appropriate use of antibiotics in patients with bacterial infections, with no impact on mortality in patients with viral infections. If more accurate data on CFRs were available our model could easily be re-run, although we suspect the principle findings would remain unchanged.

We agree that it would be interesting to perform different analyses for children and adults but currently we do not have the data with which to do this.

We have updated the manuscript to clarify that whilst multiplexed PCR platforms require less training and infrastructure than standard platforms, they cannot resolve all challenges associated with performing diagnostic microbiology in resource-constrained settings.

We are aware of the important study by Keitel and colleagues that incorporates clinical signs and point-of-care tests to improve management of febrile illness in resource-limited settings. Whilst, to our knowledge, they did not explore the dynamic incorporation of aetiological surveillance data as we describe here, both their group and ours are currently undertaking prospective studies to explore the utility of this approach.

Asymptomatic parasitaemia is unlikely to be responsible for a substantial number of positive malaria RDTs in our low-prevalence setting, and we agree with the reviewer that this issue need not be addressed here.

Unfortunately, we are not aware of any reliable granular data from the Savannakhet region that could be used to validate our mortality projections.

Short courses of tetracyclines are regularly used to treat paediatric scrub typhus in our settings, without an increase in adverse events. As the reviewer eludes to, tetracyclines are contraindicated in pregnancy, and although the evidence base is limited alternative treatments for scrub typhus in pregnant women, for example azithromycin, may be preferable. However, incorporating this into our model is beyond the scope of the current analysis.

# Peer review: Reviewer response for version 1

**DOI:** 10.21956/wellcomeopenres.16335.r34608

**Published:** 2019-02-15

**Authors:** Tamer H. Farag

**Affiliations:** 1 Institute for Health Metrics and Evaluation (IHME), Seattle, WA, USA

Chandna and colleagues have compared the performance of surveillance-guided and biomarker-guided antibiotic therapy, both separately and combined, in computer model simulations.  A strength of this exercise, is that the authors parameterized the model using estimates based on data collected largely from the Savannakhet region, where the simulation is set.  They simulated performance both in terms of DALYS averted and cost-savings per DALY.  The authors also thoroughly described the potential limitations of the model, addressing many of my concerns as a reviewer.

 On page 8 they stated that "our aim is only to determine whether this approach warrants further investigation in a real-life setting, and not provide a robust estimate of potential magnitude of its impact", which succinctly describes the elegance of this exercise and others like it.  It would have been even better had this aim been stated in the abstract and in the introduction, so that readers can properly contextualize the methods and results sections.

 While a well-conducted, well-explained study, there was one element that I thought was a potential drawback that ought to be addressed in the discussion. The authors used local hospital presentations to represent population-based epidemiology and (in the simulation) improve upon standard of care. They then concluded that antibiotic prescribing based on regional surveillance can improve upon standard of care. There are two issues with this:

 1) Hospital presentations do not represent cases in the general population, tending to be comprised of more serious cases that have been referred up the clinical chain, minus fulminant cases that die before they can present in hospital.

 2) Being conducted in one region, they cannot say that their regional epidemiology differs from region to region. Rather, it may simply be the case that the standard of care is wrong and should be altered everywhere to match more closely to their findings from Savannakhet.

 The authors have been so thorough with their caveats that I suspect the above may have been addressed in a tangential way that I did in fact miss.  However, I do think the discussion section ought to explore the utility of population-based surveillance for informing community-delivered care. Population-based surveillance can both determine regional variation, and population (not hospital) incidence of infections causing febrile illness. 

 Two such initiatives are the Child Health and Mortality Prevention Surveillance (CHAMPS) program, which uses pathology-based methods to accurately determine cause of death among children in demographic surveillance sites, and the Countrywide Mortality Surveillance for Action (COMSA) platform, a sample registration system that is designed to draw inference from CHAMPS. Paired with disease mapping initiatives such as those run from my institution, we may be able to provide enough locally-specific inference to inform efforts such as the ones simulated by Chandna and colleagues.

 To conclude, this study appears to be methodologically sound, well-reasoned, clearly-presented and well-written. In addition, it is a wonderful example of value that can be added to previously collected primary data through the use of simulation. It is a valuable addition to the literature as the global health community grapples with what it means to provide "precision public health", or healthcare that is matched to to locally-specific estimates.

I confirm that I have read this submission and believe that I have an appropriate level of expertise to confirm that it is of an acceptable scientific standard.

# Response

**Published:** 2019-07-30

**Authors:** Arjun Chandna

**Affiliations:** Mahidol-Oxford Tropical Medicine Research Unit, Thailand

We thank the reviewer for taking the time to read our manuscript so carefully. We are pleased that the reviewer recognises the potential merits of simulation, and how it might be leveraged to guide future research exploring alternative approaches to “precision public health”. We are equally pleased that the reviewer recognises our efforts to describe the limitations of our approach, as we firmly believe that this degree of transparency is essential in order for readers to contextualise results. Below we respond to the reviewer’s specific comments.

We have emphasised that our primary aim was to explore whether the combined approach of host biomarker- and surveillance-guided antibiotic therapy warrants further exploration by including this statement in the Introduction (as well as in the Discussion). We have not included it in the Abstract in the interests of brevity.

We agree that there is an inherent difficulty of extrapolating community epidemiology from health facility-based presentations. As the reviewer eludes to, we had acknowledged this in a tangential way, with regards to the likely difference in prescribing practices for patients presenting to hospital (who likely have more severe disease). In response to the reviewer’s comments we have made this more explicit, and in addition we have taken the opportunity to highlight some of the challenges of population-based surveillance in resource-limited settings, with regards to operational feasibility and low volume dried blood specimens.

We agree that we cannot comment on regional heterogeneity in fever aetiology from this study, and it is possible that the standard of care for community fever management throughout Laos should be altered to reflect our simulated findings from Savannakhet. However, as far as we are aware, we had not drawn this conclusion and therefore have not altered the manuscript to clarify this. Of note, predicted benefits of accounting for regional heterogeneity in fever aetiology in Laos has previously been described (White *et al*., PLoS ONE, 2012, DOI: 10.1371/journal.pone.0044545).
